# The state of adolescent immunization in Nigeria: a wake up call for all stakeholders

**DOI:** 10.11604/pamj.2019.33.294.18940

**Published:** 2019-08-13

**Authors:** Folusho Mubowale Balogun

**Affiliations:** 1 Institute of Child Health, College of Medicine, University of Ibadan, Ibadan, Nigeria; 2 University College Hospital, Ibadan, Nigeria

**Keywords:** Adolescents, immunization, vaccine preventable diseases, immunization programs

## Abstract

The number of children who survive to adolescence is increasing in Nigeria, significantly due to the success of child survival programs, with immunization as a major theme. However, the national immunization schedule in Nigeria is presently restricted to early childhood with no attention paid to immunization in adolescence. Presently, the vaccines that are readily available for adolescents include tetanus toxoid which is normally administered to pregnant women, so necessarily includes adolescent mothers; and a few research programs which offers hepatitis B vaccines. Also, there are few Nigerian adolescents who access immunization as a requirement for travelling outside the country or as a result of parental effort. Knowledge and awareness about adolescent immunization is generally poor. Nigerian adolescents have been shown to be poorly protected from tetanus, rubella and hepatitis B which are vaccine preventable. Neonatal, childhood and adult tetanus, congenital rubella syndrome, cervical cancer and hepatocellular carcinoma are just few of the diseases whose incidence can be reduced with an effective adolescent immunization program. This will also ensure that the gains of childhood immunization is concretized and socio-economic losses as a result of vaccine preventable diseases are eliminated to create a healthy and vibrant workforce. There is an urgent need to build a viable adolescent immunization program in Nigeria as adolescents represent a window of opportunity to prevent diseases which affect both the younger and older age group. This can be extended to other developing countries as well.

## Essay

Immunization is one of the important health breakthroughs of the last century and the use of vaccines is an effective tool available for prevention of infectious diseases with their associated morbidities and mortalities [1]. Vaccines are highly cost effective, efficient and can be financially sustained even by the poorest of countries [2]. Uncontrolled infectious diseases can place tremendous economic burden on countries but immunization has increased life expectancy and general quality of life, with improvement in economic productivity globally [3]. Adolescence describes children between ages 10 to 19 years and they are a significant proportion of the population in developing countries [4]. This is as a result of improvement in economic growth which also affects the quality of healthcare services, including the implementation of the components of the child survival strategies of which childhood immunization forms a part. Nigeria is the most populous African country and approximately 23% of her population are adolescents, making her the country with the largest number of adolescents in Africa [4]. However, immunization schedule in Nigeria is presently restricted to early childhood but adolescent immunization can no longer be ignored because delaying its implementation will result in loss of the achievements of childhood immunization. The Nigerian adolescents will have to battle with enormous morbidities and mortalities in future because of the break in their protection and it will require a lot of economic resources to alleviate some of the results of this neglect in future.

Presently, there are many Nigerian adolescent who were never immunized in infancy or did not complete the routine childhood immunization schedule due to the drop in childhood immunization rate in the nineties [5]. There have however been some gradual improvement in immunization uptake in the last few years in Nigeria [6], though there are still some operational challenges about vaccine availability. The relevant vaccines in adolescence include Hepatitis B, Diphtheria, Pertussis, Tetanus (all the three combined as DPT), Human Papilloma Virus (HPV), Varicella, Measles, Mumps and Rubella (MMR) vaccines. Some of these vaccines like DPT have been taken earlier in infancy but booster doses are required because the initial protection they confer begin to wane by adolescence. The importance of each vaccine will subsequently be discussed. Nigeria is a hyper-endemic region for Hepatitis B infection with an estimated 18 million people living with the infection [7]. The devastating result of hepatitis B infection include mortality from fulminant acute infection, becoming a chronic carrier who can infect others and also develop cirrhosis or hepatocellular carcinoma. The younger a child is at the time of infection with hepatitis B, the higher the chance of having HBeAg which indicate active carrier state [7]. The prevalence of HBeAg carrier rate reported from studies in Nigeria is between 3.3-19.2% with a higher rate in age group 0-20 years [7, 8]. Mothers with HBeAg also can infect their babies who are also prone to becoming chronic carriers. Hepatocellular carcinoma occasionally occur in childhood and a review over a 35 year period in a Nigerian tertiary health facility showed that it constituted 0.45% of abdominal malignancies seen in childhood [7]. Adults with hepatocellular carcinoma usually present late to hospitals in Nigeria when only palliative care could be rendered and this results in high mortality [8]. Preventive measures for Hepatitis B infection which includes immunization will preserve the future of Nigerian adolescents.

The second peak for pertussis infection in children apart from early infancy is 11-18 years when immunity from earlier vaccination would have waned [9]. Pertussis does not usually result in fatal outcome in adolescents but it can be unrecognized for a long time and the patient can infect unprotected young infants who have the highest risks of complications and deaths from the disease. This late diagnosis in adolescents is common and it is easily missed even in the best of centers. Booster doses of pertussis vaccine are presently not being given in Nigeria and so the prevalence of pertussis is likely to be high among Nigerian adolescents. A report from the United States showed that the odd ratio for infants of mothers aged 15-19 years developing pertussis was 7.4 if the mother has cough for more than one week [9]. Tetanus is another vaccine preventable disease which can be dangerous to the health of adolescents because of the associated high mortality. The required booster doses are not being given in Nigeria so children who completed the routine childhood immunization can still die of tetanus. Many pregnant adolescents commit abortion because they were not prepared to keep their pregnancies. In a Nigerian study, 54.7% of adolescent girls did not have antibody against tetanus and 26.8% of these had a history of unsafe abortions exposing them to post abortion tetanus [10, 11]. Many adolescent mothers in the country do not receive antenatal care from health facilities and some commence such care late, predisposing both the mother and unborn child to tetanus [12]. A review of pediatric emergency admissions in a tertiary health institution in southwest Nigeria revealed that Tetanus accounted for 32.6% of mortalities over a 35 year period and 61.6% of these were in neonates [13]. The male adolescents should also be immunized as studies showed that they are actually more prone to tetanus since they don't have the privilege of being immunized unlike their female counterparts who have access to tetanus immunization during antenatal care [14]. Male adolescents are predisposed to having tetanus infection as they engage in more endangering outdoor activities like hunting and non-mechanized methods of farming.

The sub Saharan Africa has the highest burden of Human Papilloma Virus (HPV) infection which is the main predisposing factor for the development of cervical cancer [15]. This is because the predisposing factors (which include poverty, early sexual debut, multiple sexual partners from polygamy and low socioeconomic class) are prevalent in this region. A study reported the prevalence of HPV infection to be 14.7% in rural Nigerian women [16]. This virus is sexually transmitted and persistent infection as found in chronic carriers predispose to development of cervical cancer. The earlier vaccines (Cervarix and Gardasil) protect against HPV strain 16 and 18 which are responsible for about 70% of cervical cancer [17]. The new nanovalent vaccine (Gardasil 9) protects against these earlier strains and also strains 31, 33, 45, 52 and 58 which are responsible for another 20% of cervical cancer [18]. The World Health Organization has recommended that HPV vaccine should be introduced into routine immunization in order to protect against this cancer and that in resource constrained settings, the focus should be on girls between nine to thirteen years [19]. This is to ensure girls are adequately protected before sexual debut and HPV vaccine has been shown to have best effectiveness in younger age group [20]. Compared to cervical cancer screening programs, immunization with HPV vaccine is a cost effective way of reducing the lifetime risk of cancer in these environments. Rubella is another vaccine preventable disease which is important particularly in adolescent girls. This illness is usually mild and sometimes unnoticed but with devastating consequences on the fetus (including irreversible multisystem abnormalities involving the eyes, brain, heart, ears, and intrauterine fetal death) should the mother get infected in the first trimester when the risk of mother to child transmission is about 90% [21]. Studies have shown that between 55.9 and 62% of Nigerian women are not protected from rubella [1, 21-23]. Active infection was also reported in 3.9% of pregnant women in north eastern part of Nigeria [24]. Immunity tends to increase with age as only 23.8% were immune in the 14-19 year old group compared to 74.4% in the 30-40 year age group in this study. Thus, adolescent mothers were more predisposed to having babies with congenital rubella syndrome compared to the older women. Rubella is still not a reportable disease in Nigeria, and it may be because it is generally a mild disease but the catastrophic outcome in newborns of mothers infected in the first trimester makes it an important disease to be controlled. Mumps is a viral disease which many Nigerians do not see as a serious illness as affected children appear otherwise healthy except for the swollen jaws and mild discomfort. Mumps orchitis is a complication from mumps in boys resulting in painful swelling of the testes and can lead to testicular atrophy [25]. This can cause azoospermia (total absence of sperm cells), oligospermia (low sperm count) or asthenospermia (abnormal sperm movement) which can result in subfertility or infertility [25]. There is an increasing awareness now that men equally contribute to infertility as women and the percentage of men's infertility from mumps orchitis in Nigeria is not known. Mumps can also cause meningitis and inflamed ovaries (though this is not associated with infertility).

Few Nigerian adolescents presently have the opportunity of receiving any form of immunization. These include adolescents who participate in vaccine trials in selected settings. Adolescents also benefit from vaccines given in the northern part of Nigeria to prevent meningitis epidemic caused by *Neisseria meningitides*. Finally, the Nigerian immunization policy makes provision for administration of Tetanus toxoid to pregnant mothers starting from 15 years of age, thus covering pregnant mid and late female adolescents. Immunization in all these settings is not structured or organized to reflect any exclusivity for adolescents. Some immunizations are listed as entry requirements into developed countries, prompting few privileged Nigerian adolescents from higher socio6economic class, seeking educational opportunities to take these vaccines. There are also adolescents whose parents are aware of such vaccines and they ensure their adolescents get them. This group is also in the minority as many Nigerian parents as well as parents in many sub-Saharan countries are unaware of availability of vaccines specifically for adolescents [26]. There are other barriers to the uptake of vaccines by Nigerian adolescents despite the availability of such vaccines in the country. These include high cost of the vaccines, lack of awareness on the part of parents, poor knowledge of health care workers about the vaccines and lack of government legislation to encourage health workers to recommend the vaccines [26, 27].

Nigeria is long overdue to have an organized adolescent health program to address the needs of her adolescents. The first step in meeting this need is to determine which vaccine preventable diseases will be selected for the program based on comparison of results of burden of diseases of public health importance that vaccines can prevent [28]. The information required can be obtained from health records and when this is absent as it is often the case in many sub-Saharan African countries, mathematical modelling can be used to estimate the disease burden. The public and medical community perception of the vaccine is also important at this stage as it will ultimately affect its acceptance and uptake [28]. Thereafter, vaccines which have shown high quality, efficacy and safety for the identified diseases will be compared and the best for the country will be determined taking into consideration financial sustainability and some economic indices like cost benefit and cost effectiveness. Apart from these policy issues, the programmatic aspect of vaccine introduction is also important to ensure the success of adolescent immunization program [28]. The form and presentation of the selected vaccine should be considered. The availability of the vaccine should be discussed with relevant bodies like the World Health Organization and United Nation Children Fund who will ensure continuous supply of the vaccine. The existing immunization structure (including manpower and equipment) will also require strengthening so as to accommodate the introduction of the vaccines. The other important steps are creation of awareness among all stakeholders, decision about the mode of vaccine introduction and monitoring and evaluation after the commencement of the program. The peculiarity of Nigeria has to be taken into consideration to ensure a successful adolescent immunization. In determining how the target population can be reached for example, community based adolescent immunization program may be better in Nigeria because a great number of adolescents are out of school. This is unlike Uganda and Kenya where HPV vaccine was introduced in schools and a high coverage was obtained because school enrollment was high [29]. Nigeria also had a history of boycott of Polio vaccine which became a global concern [30]. This was as a result of suspicion that the polio vaccine was a fertility control measure and some also believed it was contaminated with HIV. Targeting only females for immunization as it is being done for HPV and Tetanus vaccines could raise suspicions as reported from other African countries [14]. There is therefore a need for involving relevant stakeholders from the planning stage and utilizing both the traditional and modern means of communicating information about the immunization program. A well-established adolescent immunization program in Nigeria will ensure that the gain of infant immunization is not lost and will produce a healthier population of adolescents which will be of great benefit to the country socioeconomically. There are some African countries like South Africa who have demonstrated that adolescent immunization program is feasible and this is also possible in Nigeria.

## Competing interests

The author declares no competing interests.
